# Single-Molecule Fluorescence Polarization Study of Conformational Change in Archaeal Group II Chaperonin

**DOI:** 10.1371/journal.pone.0022253

**Published:** 2011-07-14

**Authors:** Ryo Iizuka, Taro Ueno, Nobuhiro Morone, Takashi Funatsu

**Affiliations:** 1 Graduate School of Pharmaceutical Sciences, The University of Tokyo, Tokyo, Japan; 2 Department of Ultrastructural Research, National Institute of Neuroscience, National Center of Neurology and Psychiatry, Tokyo, Japan; 3 Core Research for Evolutional Science and Technology (CREST), Japan Science and Technology Agency, Tokyo, Japan; Université Joseph Fourier, France

## Abstract

Group II chaperonins found in archaea and in eukaryotic cytosol mediate protein folding without a GroES-like cofactor. The function of the cofactor is substituted by the helical protrusion at the tip of the apical domain, which forms a built-in lid on the central cavity. Although many studies on the change in lid conformation coupled to the binding and hydrolysis of nucleotides have been conducted, the molecular mechanism of lid closure remains poorly understood. Here, we performed a single-molecule polarization modulation to probe the rotation of the helical protrusion of a chaperonin from a hyperthermophilic archaeum, *Thermococcus* sp. strain KS-1. We detected approximately 35° rotation of the helical protrusion immediately after photorelease of ATP. The result suggests that the conformational change from the open lid to the closed lid state is responsible for the approximately 35° rotation of the helical protrusion.

## Introduction

**Figure 1:**
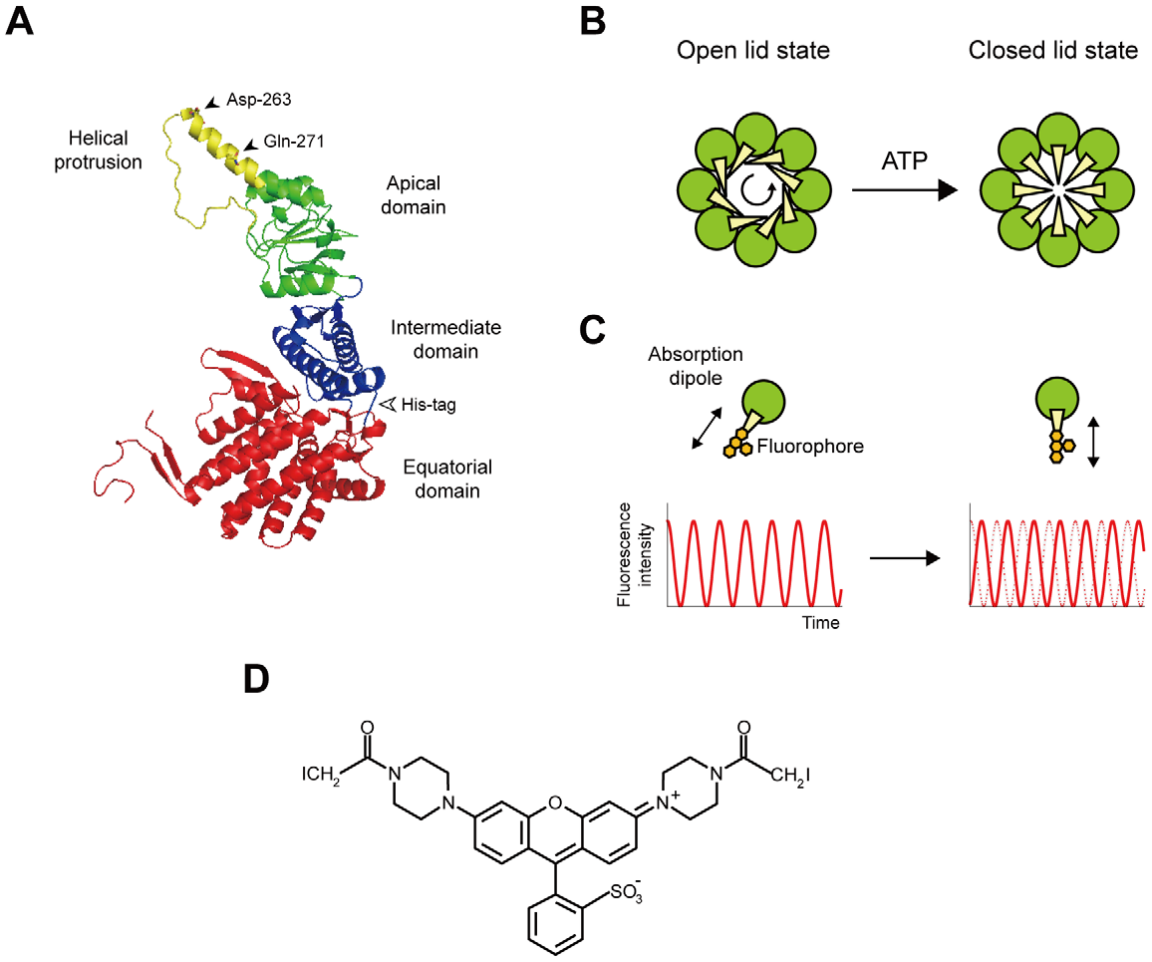
Model for the conformational transition from the open lid to the closed lid state in group II chaperonins and experimental design. (**A**) Subunit structure of *T*. KS-1 α chaperonin. The subunit has three distinct domains: equatorial (*red*), intermediate (*blue*), and apical (*green* and *yellow*). The helical protrusion is color-coded in *yellow*. Asp-263 and Gln-271 (*black arrowheads*) were mutated to cysteines, and were crosslinked with a bifunctional fluorophore. A His-tag was inserted into the loop, connecting the equatorial and intermediate domains (*white arrowhead*). The coordinates are from the Protein Data Bank code 1Q3S [12], and figures were drawn with the PyMOL program (http://pymol.sourceforge.net/). (**B**) Model for the conformational transition from the open lid to the closed lid state in the group II chaperonins [13], [14], [22]. ATP drives the conformational change from the open lid to the closed lid state. At this point, the counterclockwise rotation of the apical domains (*green*) orients the helical protrusions (*yellow*) toward the center of the cavity. (**C**) Expected intensity time courses for a single fluorophore bound to the helical protrusion. When a fluorophore is immobile, the fluorescence intensity oscillates as the excitation polarization is rotated (*red curve*). A phase shift is expected after reorientation of the fluorophore as a result of the rotation of the helical protrusion. (**D**) Structure of bis-((*N*-iodoacetyl)piperazinyl)sulfonerhodamine (BSR).

Chaperonins are ubiquitous molecular chaperones that form double-ring assemblies of about 60 kDa subunits. The resulting structure has a large central cavity in which non-native proteins can undergo productive folding in an ATP-dependent manner [1], [2]. There are two phylogenic groups of chaperonins [3], [4]: Group I chaperonins, which are present in bacteria (GroEL), mitochondria (mitochondrial 60-kDa heat-shock protein, mtHsp60), chloroplasts (Rubisco subunit binding protein, RBP), and some archaea, consist of double heptameric rings; Group II chaperonins, which are present in archaea (referred to as thermosomes) and the eukaryotic cytosol [known as TCP1 ring complex (TRiC) or chaperonin-containing TCP1 (CCT)] are assembled in two octameric or nonameric rings. The group I and II chaperonins share a similar domain arrangement [5], [6]. Each subunit comprises three distinct domains: equatorial, intermediate, and apical (Figure 1A). The equatorial domain contains the ATP binding site and is involved in intra- and inter-ring contacts. The apical domain is involved in binding to substrate proteins. The intermediate domain connects the equatorial and apical domains of each subunit, and transfers the ATP-induced conformational changes from the equatorial to the apical domain.

The most striking structural difference between the group I and II chaperonins is the lid of the central cavity of the chaperonin complex. In GroEL, the cofactor GroES interacts with one or both of GroEL rings in an ATP-regulated fashion, thereby sealing the cavity from the outside. In contrast, the group II chaperonins do not require the cofactor; the helical protrusion at the tip of the apical domain substitutes for the cofactor as a built-in lid on the central cavity [6]–[10] (Figure 1A). It has been shown that ATP drives the conformational change of the group II chaperonins, from the open lid, substrate-binding conformation to the closed lid conformation, to encapsulate unfolded protein in the central cavity [7], [8], [10]. The group II chaperonin-mediated protein folding is critically dependent on the lid closure [8], [10], [11]. Until date, most of the available structural information on the group II chaperonins has been obtained from “static” structural analyses, such as X-ray crystallography [6], [12]–[14], electron microscopy [7], [15]–[22], and small angle X-ray scattering [8], [10], [23]. To understand further how the local changes in the nucleotide-binding site lead to lid closure, it is necessary to directly observe the conformational change from an open lid to a closed lid state during the functional cycle. Therefore, we studied the rotational dynamics of the helical protrusion by single-molecule fluorescence polarization microscopy using α chaperonin from a hyperthermophilic archaeum, *Thermococcus* sp. strain KS-1 (*T*. KS-1) [9]–[11], [23]–[26]. We then demonstrated that the conformational change from an open lid to closed lid state can be achieved by approximately 35° rotation of the helical protrusion.

## Results

Polarization of light that is used to excite a single molecule provides a means to probe the orientation dynamics of individual molecules [27], [28]. We used a single-molecule polarization modulation method to probe the rotation of the helical protrusion of *T*. KS-1 α chaperonin [29]–[32] (Figure 1A–C). The principle is as follows: Under the rotating polarized excitation, fluorescence intensity from an immobile fluorophore is expected to be proportional to a quadratic cosine function (see Materials and Methods). The intensity reaches maximum and minimum when the excitation polarization becomes parallel and orthogonal, respectively, to the absorption dipole of the fluorophore, enabling the determination of the orientation of the fluorophore. Thus, the change in the orientation of the fluorophore can be detected as the change in phase of the cosine curve (Figure 1C).

For polarization studies, it is quite helpful to use a bifunctional fluorophore, which significantly reduces the fluorophore wobble. Therefore, we used a bifunctional thiol reactive rhodamine derivative, bis-((*N*-iodoacetyl)piperazinyl) sulfonerhodamine (BSR) (Figure 1D). The transition dipole moment of BSR is aligned along the long axis of three coplanar rings of the fluorophore [33]. Hence, the average orientation of the fluorescence dipole is expected to be parallel to a line joining the cysteines. In conjunction with BSR, we created the double-cysteine mutant of *T*. KS-1 α chaperonin with internal hexahistidine tags (Figure 1A). The target residues for replacement with cysteines, Asp-263 and Gln-271, are located at the tip of the helical protrusions (Figure 1A). Site-directed mutagenesis was used to prepare the mutant by substituting an endogenous cysteine at the 366^th^ position with serine, and by introducing two cysteine residues at the 263^th^ and 271^th^ positions. The double-cysteine mutant was able to undergo nucleotide-dependent conformational changes (Figure S1A). The mutant was also able to facilitate the refolding of GFP in an ATP-dependent manner despite the yield being less than half that of the wild-type (αWT) (Figure S1B). In the BSR-labeled double-cysteine mutant (BSR-CPN), the dipole of BSR was expected to be almost parallel to the axis of the helical protrusion.

**Figure 2:**
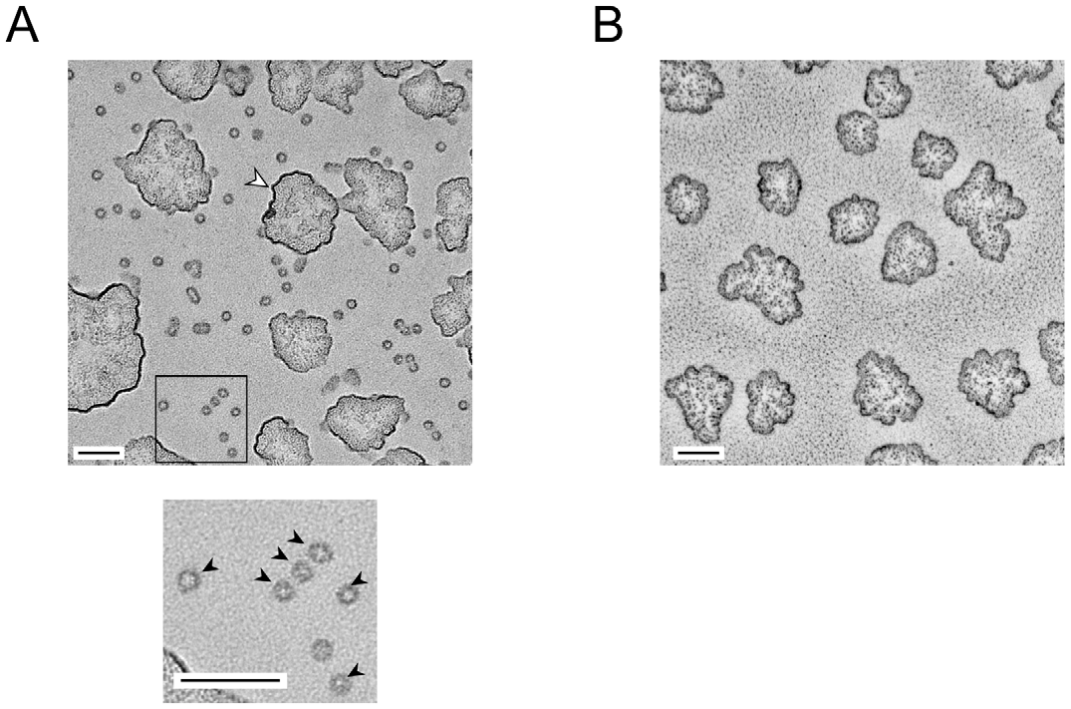
Rotary-shadowed electron micrograph of chaperonin immobilized onto the coverslip surface. (A) With immobilized chaperonin. The boxed area in the *upper panel* is enlarged in the *lower panel*. The particles comprise almost exclusively ring-shaped end-on views (*black arrowheads*). The white arrowhead indicates a cluster of glycerol. (B) Without immobilized chaperonin. The scale bars represent 100 nm.

We tested several methods to immobilize BSR-CPN suitable for single-molecule polarization modulation experiments. Among these, the direct immobilization onto the coverslip was the most effective. In addition, the coverslips were used immediately after cleaning with an oxygen plasma asher. The plasma-treated coverslips had extremely flat surfaces, whereas the commercial coverslips had patterned indented surfaces (data not shown). The orientation of BSR-CPN immobilized onto the coverslip surface was investigated with an electron microscope (Figure 2). The specimen was treated using the low-angle rotary shadowing method. On the coverslip with immobilized chaperonin, most of the single particles were found to be ring-shaped (Figure 2A, *black arrowheads*), corresponding to end-on views of the chaperonin complex. On the other hand, such particles were not observed on the coverslip without immobilized chaperonin (Figure 2B). Thus, BSR-CPN was considered to be orientationally immobilized on the glass surface because of hydrophobic interaction.

**Figure 3:**
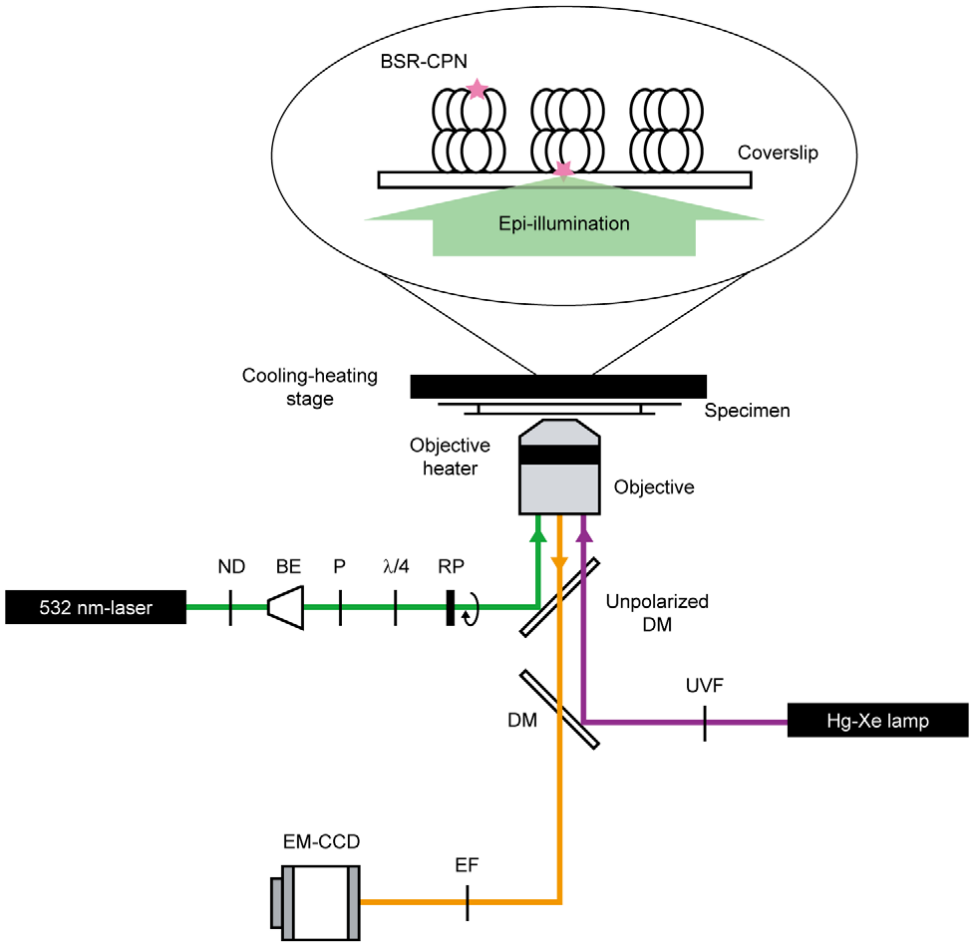
Schematic drawing of the microscopic system for modulating the excitation polarization. Optical paths for different wavelengths are distinguished by their colors. The polarization of excitation laser (*green*) was continuously modulated by a polarizer mounted on a motorized rotary holder. The polarizer was rotated at 20.01°/s. Fluorescence (*orange*) was collected through an objective, and detected with an electron multiplying charge-coupled device camera. Caged ATP was photolyzed by UV light from a Hg–Xe lamp (*purple*). BSR-CPN was directly immobilized onto the surface. ND, neutral density filter; BE, beam expander; P, polarizer; λ/4, quarter wave plate; RP, rotating polarizer; DM, dichroic mirror; EF, emission filter; UVF, UV sharp-cut filter. The figure in the ellipse connotes a specimen for microscopic observation. BSR (*pink star*) was excited using an epi-illumination configuration.

BSR-CPN immobilized on the coverslip was excited with continuously modulated polarized light (20.01°/s) using an epi-illumination configuration (Figure 3). Because *T*. KS-1 α chaperonin has reduced activity at <50°C temperatures [24]–[26], it is necessary to perform microscopic experiments at ≥50°C. Therefore, the microscopic specimen was incubated at 50°C with a cooling–heating stage and an objective heater (Figure 3). We confirmed that the temperature of the specimen reached 50°C from the thermal quenching of fluorescence (Figure S2). To our knowledge, the results presented here are the first demonstration of single-molecule fluorescence observation at 50°C. The conformational change in BSR-CPN was induced by the photorelease of ATP from caged ATP, which was photolyzed by a 500-ms pulse of UV light from a Hg–Xe lamp (Figure 3). Approximately 60% of the caged ATP (100 µM) was split to produce ATP at 23°C (data not shown). *T*. KS-1 α chaperonin incubated with approximately 60 µM ATP exists in an asymmetric conformation: one open ring and one closed ring (Iizuka et al., unpublished data).

**Figure 4:**
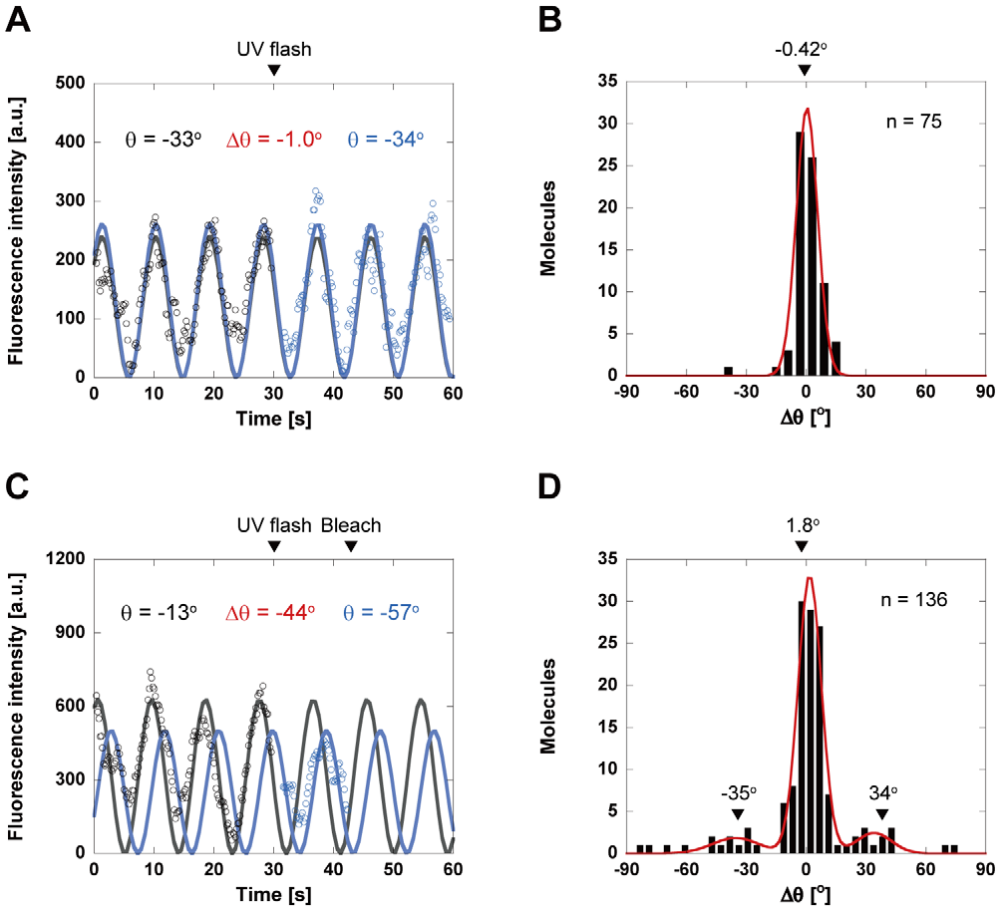
Time trajectories of the fluorescence intensity and calculated fluorophore angles. (**A**, **C**) Time trajectory of BSR intensity in the absence (**A**) and presence of caged ATP (**C**). The UV flash occurred at 30 s (*arrow*). The trajectories of BSR intensities after subtracting the background signals were averaged over every five frames, and fitted to a quadratic cosine function (*solid lines*). *Black*, fluorescence intensity before UV flash; *blue*, fluorescence intensity after UV flash. (**B**, **D**) Distributions of Δ*θ* in the absence (**B**) and presence of caged ATP (**D**). *Positive angle*, angular displacement measured counterclockwise; *negative angle*, angular displacement measured clockwise. In the absence of caged ATP (**B**), the distribution corresponded to a Gaussian distribution with a peak of −0.42°. In the presence of caged ATP (**D**), the distribution can be fitted by a sum of three Gaussian functions, with peaks of 1.8°, 34°, and −35°.

We then monitored changes in the dipole orientation of BSR attached to the helical protrusion at 50°C. Figure 4A shows a typical time trajectory of BSR fluorescence in the absence of caged ATP. The UV flash occurred at 30 s. The resulting trajectories of BSR fluorescence were fitted to a quadratic cosine function (see Materials and Methods), and the angle *θ* between the polarization axis of the excitation light and that of the fluorophore dipole was determined. The angular displacement between the values of *θ* before and after the UV flash was defined as Δ*θ*. The positive and negative angles are equivalent to the counterclockwise and clockwise rotation of the helical protrusion, respectively. The angular distribution of 75 molecules corresponded to a Gaussian function with a peak at –0.42°, reflecting the well-defined orientation of BSR in the absence of ATP (Figure 4B). Among the molecules observed in the presence of 100 µM caged ATP (136 molecules), most did not exhibit angular changes after the photorelease of ATP from caged ATP. A significant fraction of the molecules did not exhibit the angular changes, possibly because the molecules were functionally inactive as a result of the direct interaction with the hydrophobic glass surface. However, in approximately 10% of these molecules, changes in the orientation of BSR were observed immediately after photorelease of ATP (within 1 s), indicating the ATP-induced rotation of the helical protrusion (Figure 4C). A similar result has been reported in a previous single-molecule study, where the enzyme molecules are directly immobilized onto the glass surface [34]. The resulting distribution of Δ*θ*, i.e., the rotational angle of the helical protrusion, showed discrete peaks that can be fitted by a sum of three Gaussian functions with peaks of 1.8°, 34°, and −35° (Figure 4D). Considering the previous findings that the apical domain and helical protrusion rotate counterclockwise [13], [14], [22], a rotation through a positive angle of 34° is considered to be a counterclockwise rotation of the helical protrusion within the upper ring (Figure 3). A negative angular displacement of −35° would correspond to the counterclockwise rotation of the helical protrusion within the lower ring (Figure 3).

## Discussion

**Figure 5:**
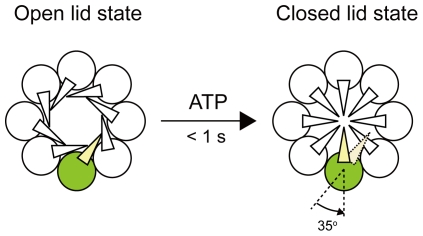
Conformational change of *T*. KS-1 α chaperonin subunits. ATP induces the conformational change from the open lid to the closed lid state, which is responsible for a approximately 35° counterclockwise rotation of the helical protrusion.

For further evaluation of the conformational change from the open lid to the closed lid state, we used a single-molecule to probe the rotation of the helical protrusion of the archaeal group II chaperonin. Our observation showed that the helical protrusion rotates approximately 35° to seal off the central cavity (Figure 5). Recently, three high-resolution structures of the group II chaperonins in the open lid state have been determined [13], [14], [22]. The apical domain and helical protrusion are found to rotate 30°–40° in a counterclockwise direction relative to the closed lid state. Our observations complement these structural studies. Interestingly, Pereira *et al.* and Zhang *et al.* indicated that three domains of each subunit reorient as a single rigid body, undergoing a counterclockwise rotation in the group II chaperonins [13], [22]. There is a completely distinct closing mechanism in the group II chaperonins as compared with the group I chaperonins. In a GroEL–GroES complex, GroES caps GroEL rings, and large structural changes are observed in GroEL. The apical domain rotates 90° along its axis and 60° upwards, and the intermediate domain closes down at approximately 25° to the equatorial domain [35]. This difference between the two groups is not really surprising, because the group II chaperonins are independent of the GroES-like cofactor.

It is believed that the domain rotation alone is not enough to complete the lid closure. Kanzaki *et al.* suggested that ATP causes the independent conformational change of the subunit, and further structural transition for the complete closure of the lid is induced and stabilized by the interaction between the helical protrusions [11]. As a result, the cavity switches from a hydrophobic to hydrophilic environment, where productive folding occurs [6], [12], [13], [14].

X-ray crystallography and cryo-electron microscopy provide high spatial resolution, but they cannot measure changes in a functioning sample over time. Spectroscopy and small-angle X-ray scattering of bulk samples have temporal resolution, but are difficult to quantify and interpret structurally because averaging among unsynchronized populations of molecules reduces the signal changes. Polarization-analyzed single-molecule imaging is a powerful tool for monitoring real-time conformational changes in proteins. Our experimental system, in which the specimen temperature is controllable, should be useful for elucidating conformational changes in other proteins at single-molecule resolution.

## Materials and Methods

### Materials and reagents

Apyrase, glucose oxidase from *Aspergillus niger*, and catalase from bovine liver were obtained from Sigma. Bis-((*N*-iodoacetyl)piperazinyl)sulfonerhodamine (BSR) and adenosine 5′-triphosphate, *P* ^3^-(1-(4,5-dimethoxy-2-nitrophenyl)ethyl) ester, and disodium salt (caged ATP) were purchased from Invitrogen. Caged ATP was treated with apyrase before use to remove contaminating ATP. Other reagents were obtained from Wako Pure Chemicals. Coverslips were obtained from Matsunami Glass. The coverslips were placed in an oxygen plasma asher (FEMTO; Diener electronic) to clean their surfaces before use.

### Construction of chaperonin mutant

For insertion of the hexa-histidine sequence between amino acids at positions 146 (Val) and 147 (Asp) of *T*. KS-1 chaperonin α subunit (αWT-His), a two-step PCR was performed. The N-terminal fragment (amino acids 1–146) was amplified with the following primer sets: primer A [5′- GGAATTCCATATGGCACAGCTTAGTGGACAG-3′ (*Nde* I site underlined)]; primer B (5′-CAACCCTTATGGCTATCTCGTC-3′). Similarly, a C-terminal fragment (amino acids 142–548) was amplified by primer C [5′- ATAGCCATAAGGGTT **CACCATCACCATCACCAT**GACCCGGACGACGAGGAGACCC-3′ (regions encoding amino acids 142–146 underlined)] to incorporate six histidine residues (bold) and a region to overlap with the N-terminal fragment, and primer D [5′-CGGAATTCTCACATGCCCATGTCCATTCC-3′ (*Eco*R I site underlined)]. PCR amplification was conducted with KOD –Plus– (Toyobo) using the plasmid pK1Eα2 as a template [25]. The amplified fragments were purified by agarose gel electrophoresis and used together to amplify the entire modified α subunit using primers A and D. The amplified fragment was excised with *Nde* I/*Eco*R I, and then introduced into the *Nde* I/*Eco*R I-digested pET23b vector. The resultant plasmid was designated pTKSαWT-His.

The double-cysteine mutant, αD263C/Q271C/C366S-His, was obtained using the QuikChange site-directed mutagenesis kit (Stratagene) using pTKSαWT-His as a template. The oligonucleotides used for mutagenesis are listed in Table S1.

### Purification of chaperonins

Chaperonin complexes with hexahistidine tags were overexpressed in *E. coli*, as described previously [9]. The harvested cells were suspended in buffer A (25 mM HEPES-NaOH (pH 7.5), and 500 mM NaCl) and disrupted by sonication on ice. The supernatant after centrifugation (25,000 g, 60 min, 4°C) was applied to a HiTrap chelating HP column (GE Healthcare UK Ltd.) equilibrated with buffer A. Proteins were eluted with a linear gradient of 20–400 mM imidazol in the same buffer. MgCl_2_, glycerol, and dithiothreitol (DTT) were added to the fractions containing chaperonin complexes to 25 mM, 5% (v/v), and 1 mM, respectively, and the mixture was subjected to heat treatment at 70°C for 30 min. After denatured proteins were removed by centrifugation (25,000 g, 30 min, 4°C), the supernatant was concentrated by ultrafiltration. The concentrated fractions were loaded onto a gel filtration column (HiLoad 26/60 Superdex 200 prep grade; GE Healthcare UK Ltd) equilibrated with Buffer B [20 mM HEPES-NaOH (pH 7.5), 5 mM MgCl_2_, 150 mM NaCl, and 1 mM DTT]. Chaperonin complexes without hexahistidine tags were expressed and purified as described previously [9]. Purified chaperonin complexes were concentrated by ultrafiltration, and stored in 20% (v/v) glycerol at −80°C before use. Their concentrations were determined with a Bradford assay kit (Bio-Rad) using bovine serum albumin as the standard, and are expressed as molar concentrations of hexadecamer in this study.

### Rotary shadowing

The flow chamber was constructed from a bottom coverslip (18 mm ×18 mm) and a top coverslip (4.5 mm ×4.5 mm) separated by two spacers made of a coverslip. αD263C/Q271C/C366S-His (50 µg/mL) was directly immobilized on the surface of the coverslips. The chamber was washed with three volumes of HKM buffer [25 mM HEPES-KOH (pH 7.4), 100 mM KCl, and 5 mM MgCl_2_], and then with three volumes of 50% (v/v) glycerol. The chamber without immobilized chaperonins served as a negative control. The top coverslips were dried by vacuum evaporation, and shadowed at an angle of 5° with platinum and carbon (BAF060; Bal-Tec and EM-19500 JFDII; JEOL). The replicas were detached from the coverslip with hydrofluoric acid, washed with distilled water, applied to electron microscope specimen grids, and observed under a transmission electron microscope (Tecnai G^2^ Spirit; FEI Company and JEM-1400; JEOL) at an accelerating voltage of 120 kV. Images were recorded by a charge-coupled device camera (Ultrascan; Gatan and Veleta; Olympus Soft Imaging Solutions) at a magnification of 50,000.

### Sample preparation for microscopy

The double-cysteine mutant was labeled with BSR in HK buffer [25 mM HEPES-KOH (pH 7.4) and 100 mM KCl]. The labeled double-cysteine mutant (BSR-CPN) was separated from unreacted reagents by a NAP-5 column (GE Healthcare UK Ltd.) equilibrated with HK buffer. Labeling resulted in a stoichiometry of approximately 0.3 BSR dye molecules per chaperonin hexadecamer to reduce multiple labeling. The labeled proteins were flash frozen in liquid nitrogen and stored at −80°C until use.

The flow chamber was constructed from a bottom coverslip (25 mm ×60 mm) and a top coverslip (18 mm ×18 mm) separated by two spacers of 50 µm thickness (Lumirror #50-S10; Toray Industries Inc.). BSR-CPN was directly immobilized on the surface of the coverslips. Experimental results shown in this paper were obtained in HKM buffer containing an oxygen scavenger system (25 mM glucose, 50 units/mL glucose oxidase, 50 units/mL catalase, and 10 mM DTT) and 100 µM caged ATP. The specimen was used for microscopic observation after a 5-min incubation at 23°C to reduce molecular oxygen from the solution prior to observation (Figure S3).

### Microscopic system for modulating the excitation polarization

A schematic drawing of the microscopic system for modulating the excitation polarization is shown in Figure 3. The surface-immobilized BSR-CPN was excited using an epi-illumination configuration with a 532-nm laser (approximately 1.5 mW, COMPASS315M-100; Coherent) through an oil-immersion objective (PlanApo 100×, NA 1.4; Olympus). The incident beam from the laser was passed through a neutral density filter (Sigma Koki), a beam expander (Sigma Koki), a polarizer (Sigma Koki), a quarter-wave plate (Suruga Seiki), and a rotating polarizer, and the beam was focused on the back focal plane of the objective lens using a focusing lens and a mirror. The polarizer was rotated at 20.01°/s by a motorized rotary holder (Suruga Seiki). Fluorescence was collected through an objective on a microscope (IX-70; Olympus) equipped with a custom-made unpolarized dichroic mirror (Shigma Koki) and emission filters (593DF40; Semrock). For photolysis of caged ATP, the specimens were illuminated by a 500-ms pulse of UV light from a Hg–Xe lamp (UVF-203S; San-Ei Electric Co., Ltd.) after passing through a UV sharp-cut filter (UTF-50S-30U; Sigma Koki). Approximately 60% of the caged ATP was split to produce ATP. Fluorescence images were captured every 200 ms for 1 min with an electron multiplying charge-coupled device camera (C9100-13; Hamamatsu Photonics). Observations were carried out at 50°C using a cooling–heating stage (LK-600PM; Japan High Tech Co., Ltd., Japan) and an objective heater (Tokai Hit Co., Ltd.).

### Data analysis

The recorded images were analyzed using a homemade program on a Halcon image processor (MVTec Software GmbH) to obtain the time trajectories of BSR fluorescence intensity. The trajectories before and after UV flash were averaged over every five frames, and were fitted using the following equation:
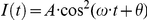



Where, *I*(*t*) is the fluorescence intensity at time *t*, *A* is the amplitude, *ω* is the angular rate of rotation (20.01°/s), and *θ* is the orientation of BSR. Rotational angles of the helical protrusion (Δ*θ*) were determined by subtracting the value of *θ* after UV flash from the value of *θ* before UV flash. The possible angles are ambiguous; they can be α° and (180 − α)°. Based on previous structural studies [13], [14], [22], the smaller angles were adopted. Data fitting was carried out using the Kaleidagraph program (version 3.6, Synergy Software).

## Supporting Information

Figure S1 **Characterization of chaperonin mutants.** (**A–C**) Protease sensitivity assay. αWT, αWT-His, and αD263C/Q271C/C366S-His (50 nM) were preincubated with or without 1 mM of the different nucleotides (ATP, AMP-PNP, and ADP) for 10 min at 60°C. Digestion with thermolysin (1 ng/µL) was carried out for 10 min at 60°C. The reaction mixtures were precipitated using 30% (w/v) trichloroacetic acid, and then analyzed on 12% polyacrylamide gels containing SDS and stained with Coomassie brilliant blue. Lane M, molecular weight marker; lane 1, without addition of thermolysin; lane 2, without addition of nucleotides; lane 3, incubated with ATP; lane 4, incubated with AMP-PNP; lane 5, incubated with ADP. (**D**) GFP refolding assay. The recovery of GFP fluorescence was continuously monitored at 510 nm at 60°C. At 0 min, acid-denatured GFP (5 µM) was diluted 100-fold in the folding buffer containing 100 nM chaperonins (*red circles*, αWT; *blue circles*, αWT-His; *yellow circles*, αD263C/Q271C/C366S-His). At 5 min after the dilution, 1 mM ATP was added. Spontaneous refolding of GFP was observed upon dilution of denatured GFP into the folding buffer without chaperonins (*black circles*). The amount recovered is expressed as a percentage of the fluorescence intensity of native GFP.
(TIF)

Figure S2 **The dependence of the fluorescence intensity on temperature.** (**A**) Relative fluorescence intensity of BSR as a function of temperature. The fluorescence intensity at 23°C was taken as 100. The change in fluorescence intensity is well fitted to a single exponential function (*solid line*). *Inset*, fluorescence spectra of BSR at 20°C −70°C. (**B** and **C**) Distributions of fluorescence intensity from single BSR molecules. The surface-immobilized BSR-CPN was observed by epifluorescence microscopy at 23°C and 50°C. The distributions of fluorescence intensity at 23°C (**B**) and 50°C (**C**) were fitted with a single Gaussian function (*solid lines*). The average intensity at 23°C and 50°C (*arrows*) are estimated to be 170 and 86, respectively.
(TIF)

Figure S3 **Distributions of time before photobleaching of a single BSR molecule.** The surface-immobilized BSR-CPN was observed by epifluorescence microscopy at 23°C and 50°C. The distributions of time before photobleaching of single BSR molecules at 23°C (**A**) and 50°C (**B**) were fitted with a single exponential function (*solid lines*), which yields the rate constants of 0.052 s^−1^ and 0.051 s^−1^, respectively.
(TIF)

Table S1 **Primer sequences used for mutagenesis.**
(DOC)
