# Supplementary figures and images for: Single-Molecule Fluorescence Polarization Study of Conformational Change in Archaeal Group II Chaperonin
Source: PLoS One. 2011 Jul 14;6(7):e22253. doi: 10.1371/journal.pone.0022253 (PMC3136518; doi:10.1371/journal.pone.0022253)

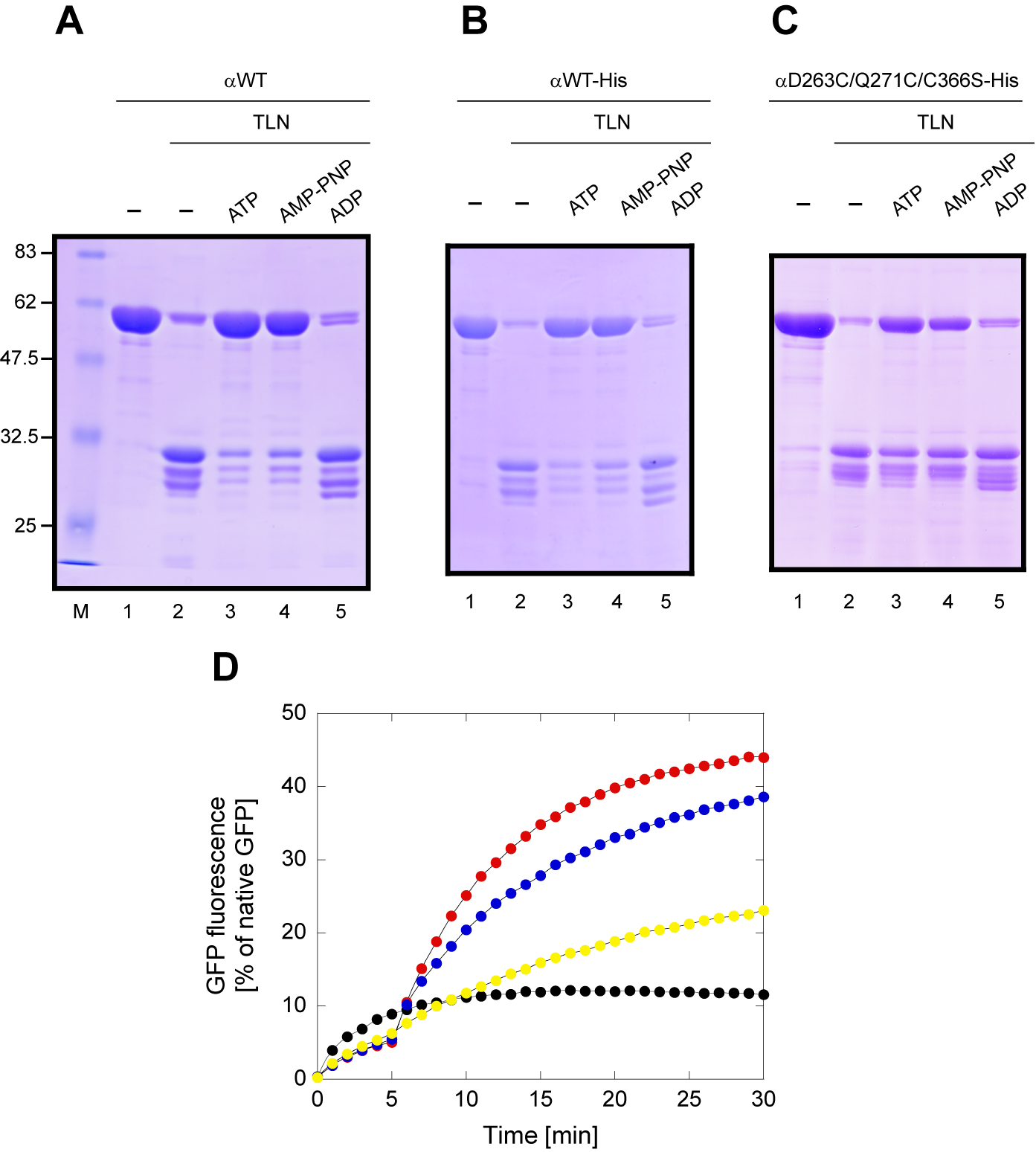

Supplement: Figure S1 — Characterization of chaperonin mutants. (A–C) Protease sensitivity assay. αWT, αWT-His, and αD263C/Q271C/C366S-His (50 nM) were preincubated with or without 1 mM of the different nucleotides (ATP, AMP-PNP, and ADP) for 10 min at 60°C. Digestion with thermolysin (1 ng/µL) was carried out for 10 min at 60°C. The reaction mixtures were precipitated using 30% (w/v) trichloroacetic acid, and then analyzed on 12% polyacrylamide gels containing SDS and stained with Coomassie brilliant blue. Lane M, molecular weight marker; lane 1, without addition of thermolysin; lane 2, without addition of nucleotides; lane 3, incubated with ATP; lane 4, incubated with AMP-PNP; lane 5, incubated with ADP. (D) GFP refolding assay. The recovery of GFP fluorescence was continuously monitored at 510 nm at 60°C. At 0 min, acid-denatured GFP (5 µM) was diluted 100-fold in the folding buffer containing 100 nM chaperonins (red circles, αWT; blue circles, αWT-His; yellow circles, αD263C/Q271C/C366S-His). At 5 min after the dilution, 1 mM ATP was added. Spontaneous refolding of GFP was observed upon dilution of denatured GFP into the folding buffer without chaperonins (black circles). The amount recovered is expressed as a percentage of the fluorescence intensity of native GFP. (TIF) [file pone.0022253.s001.tif]

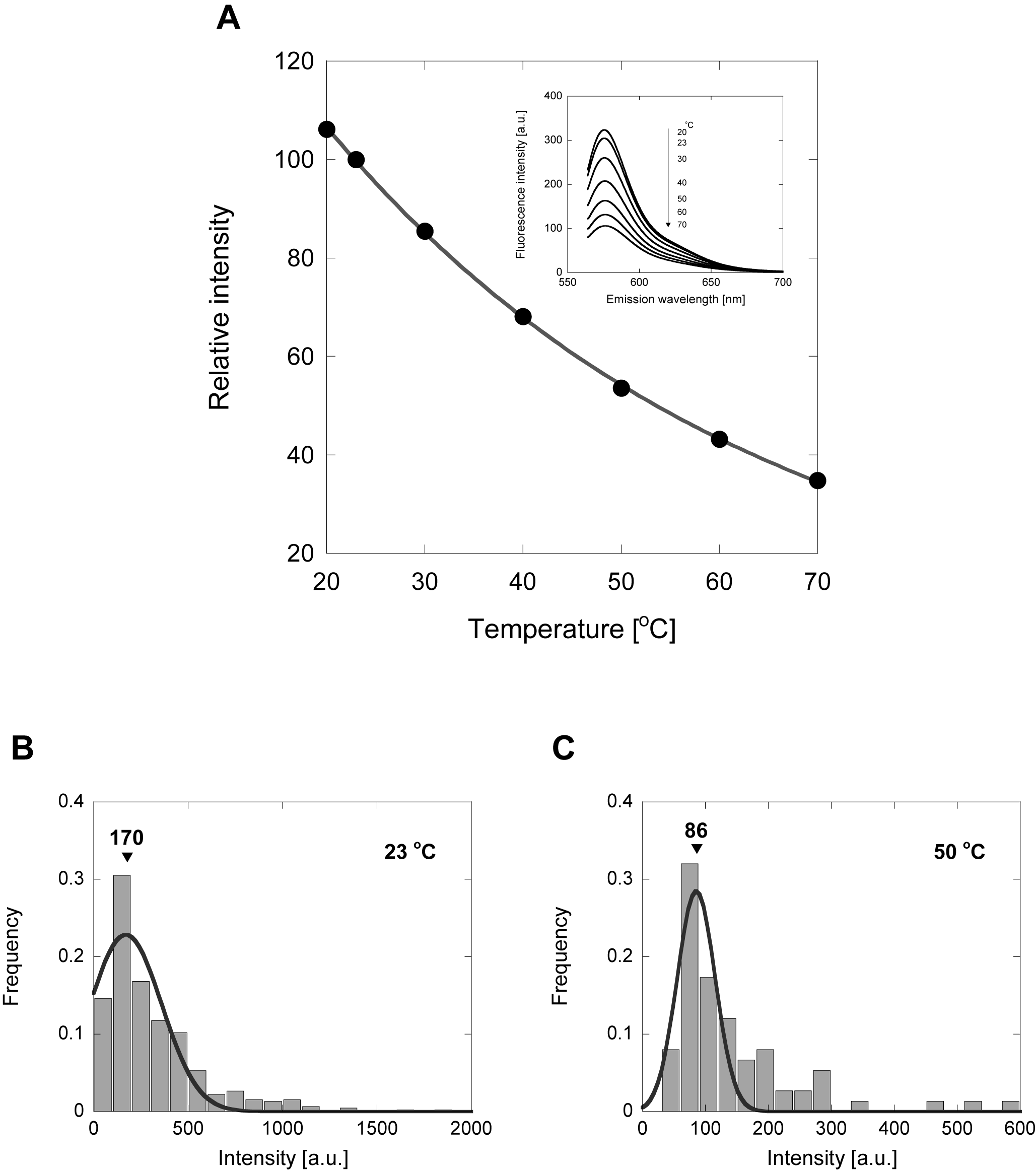

Supplement: Figure S2 — The dependence of the fluorescence intensity on temperature. (A) Relative fluorescence intensity of BSR as a function of temperature. The fluorescence intensity at 23°C was taken as 100. The change in fluorescence intensity is well fitted to a single exponential function (solid line). Inset, fluorescence spectra of BSR at 20°C −70°C. (B and C) Distributions of fluorescence intensity from single BSR molecules. The surface-immobilized BSR-CPN was observed by epifluorescence microscopy at 23°C and 50°C. The distributions of fluorescence intensity at 23°C (B) and 50°C (C) were fitted with a single Gaussian function (solid lines). The average intensity at 23°C and 50°C (arrows) are estimated to be 170 and 86, respectively. (TIF) [file pone.0022253.s002.tif]

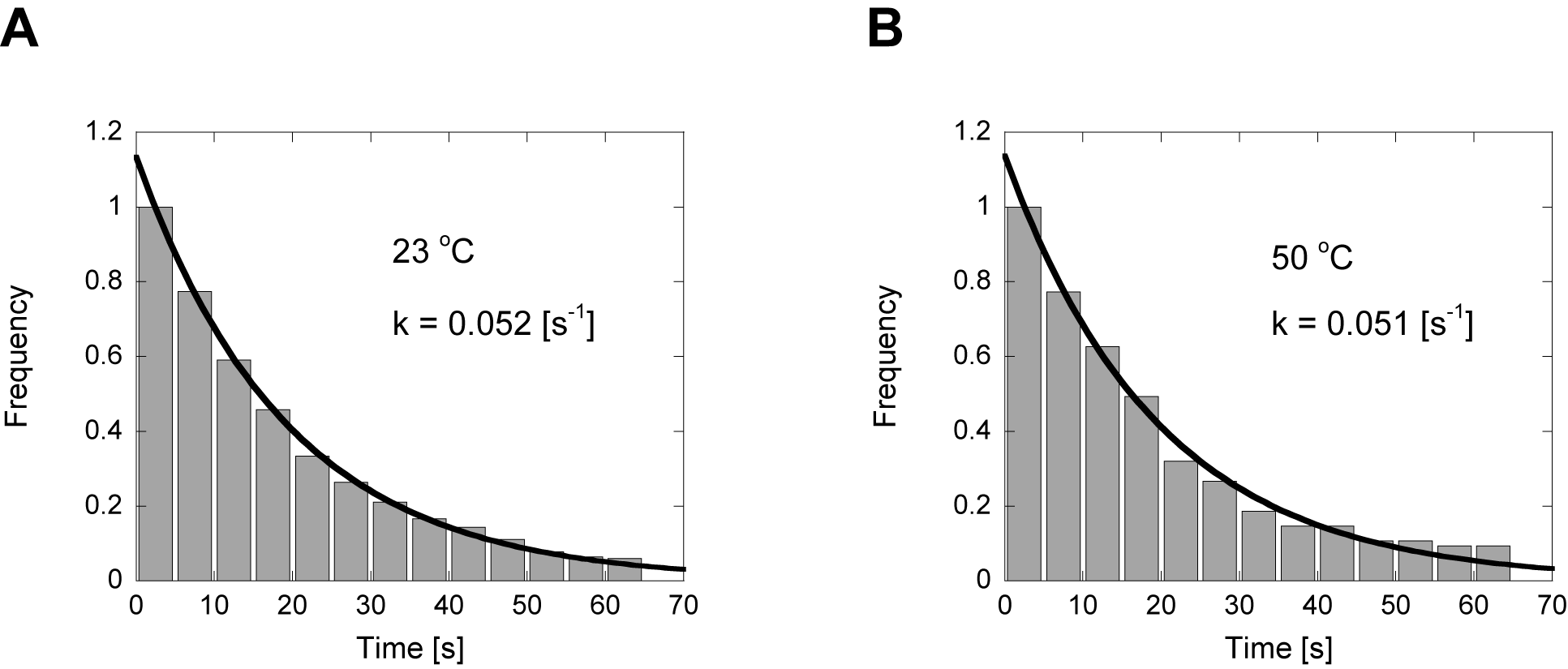

Supplement: Figure S3 — Distributions of time before photobleaching of a single BSR molecule. The surface-immobilized BSR-CPN was observed by epifluorescence microscopy at 23°C and 50°C. The distributions of time before photobleaching of single BSR molecules at 23°C (A) and 50°C (B) were fitted with a single exponential function (solid lines), which yields the rate constants of 0.052 s−1 and 0.051 s−1, respectively. (TIF) [file pone.0022253.s003.tif]
