# Supplementary material for: Single-Molecule Fluorescence Polarization Study of Conformational Change in Archaeal Group II Chaperonin
Source: PLoS One. 2011 Jul 14;6(7):e22253. doi: 10.1371/journal.pone.0022253 (PMC3136518; doi:10.1371/journal.pone.0022253)
Supplement: Table S1 — Primer sequences used for mutagenesis. (DOC) [file pone.0022253.s004.doc]

**Table S1. Primer sequences used for mutagenesis.**

| **Primer name** | **Sequence** |
| --- | --- |
| αD263C (+) | 5′-AACATAACCAGCCCGTGCCAGCTCATGAGC-3′ |
| αD263C (−) | 5′-CTCATGAGCTGGCACGGGCTGGTTATGTTG-3′ |
| αQ271C (+) | 5′-GAGCTTCCTTGAGTGCGAGGAGAAGATGC-3′ |
| αQ271C (−) | 5′-GCATCTTCTCCTCGCACTCAAGGAAGCTC-3′ |
| αC366S (+) | 5′-CTTCGTTGAGGGATCCAAGAACCCGAAGGC-3′ |
| αC366S (−) | 5′-GCCTTCGGGTTCTTGGATCCCTCAACGAAG-3′ |
